# Membrane engineering of *S. cerevisiae* targeting sphingolipid metabolism

**DOI:** 10.1038/srep41868

**Published:** 2017-02-01

**Authors:** Lina Lindahl, Aline X. S. Santos, Helén Olsson, Lisbeth Olsson, Maurizio Bettiga

**Affiliations:** 1 Department of Biology and Biological Engineering, Division of Industrial Biotechnology, Chalmers University of Technology, Gothenburg, Sweden; 2 Department of Biochemistry, University of Geneva, Geneva, Switzerland; 3 Friedrich Miescher Institute for Biomedical Research, Basel, Switzerland

## Abstract

The sustainable production of fuels and chemicals using microbial cell factories is now well established. However, many microbial production processes are still limited in scale due to inhibition from compounds that are present in the feedstock or are produced during fermentation. Some of these inhibitors interfere with cellular membranes and change the physicochemical properties of the membranes. Another group of molecules is dependent on their permeation rate through the membrane for their inhibition. We have investigated the use of membrane engineering to counteract the negative effects of inhibitors on the microorganism with focus on modulating the abundance of complex sphingolipids in the cell membrane of *Saccharomyces cerevisiae*. Overexpression of *ELO3*, involved in fatty acid elongation, and *AUR1*, which catalyses the formation of complex sphingolipids, had no effect on the membrane lipid profile or on cellular physiology. Deletion of the genes *ORM1* and *ORM2*, encoding negative regulators of sphingolipid biosynthesis, decreased cell viability and considerably reduced phosphatidylinositol and complex sphingolipids. Additionally, combining *ELO3* and *AUR1* overexpression with *orm1*/*2Δ* improved cell viability and increased fatty acyl chain length compared with only *orm1*/*2Δ*. These findings can be used to further study the sphingolipid metabolism, as well as giving guidance in membrane engineering.

Industrial processes based on microbial conversion have gained increased attention in the transformation to a more sustainable society. Microbial bioprocesses often have advantages over traditional chemical processes; for example, they make use of renewable feedstock, the catalysis conditions are more environmentally friendly, and it may be possible to produce new chemicals, difficult to synthesize using traditional chemistry^1^. A common limitation of microbial conversion is the inherent difficulty of the fermenting microorganism to maintain high productivity when conditions are perturbed^2^,^3^. For example, compounds present in the feedstock or that are produced during fermentation can negatively interfere with the physiology and key biochemical pathways of the microorganism^4^. One mode of inhibition through which these compounds can act is via interactions with biological membranes and change physicochemical properties of the membranes. A major problem in butanol production, for example, is the interaction of butanol with the plasma membrane of the fermenting organism, causing increased membrane fluidity, in turn leading to cofactor leakage and loss of membrane potential^5^. Ethanol has a similar mode of inhibition to that of butanol, although it is not as detrimental^6^. Inhibition by weak acids, on the other hand, is indirectly dependent on the plasma membrane lipid composition as they enter the cell mainly by passive diffusion over the plasma membrane, and inhibit vital functions once inside the cell^7^,^8^.

It may be possible to reduce or prevent inhibition due to molecules interacting with the cell membrane by engineering the lipid composition of the membrane. The permeability of the membrane could also be altered to reduce the uptake of inhibitory molecules to prevent damaging effects inside the cell. This concept, referred to as membrane engineering, is an attractive tool in cell factory design^3^, although only a limited number of membrane engineering projects have been described in the literature. In a study on *Saccharomyces cerevisiae*, it was shown that increasing the fraction of oleic acid (C_18:1_ fatty acid) in the plasma membrane improved the viability of the microorganism in the presence of inhibitory levels of ethanol, propanol and butanol^9^. The same strategy has also been used to increase the viability of yeast in the presence of acetic acid^10^. In another study, genetic modifications leading to enriched trans unsaturated fatty acids in the plasma membrane of *Escherichia coli*, improved its tolerance to octanoic acid as well as many alcohols, aromatic solvents and organic acids^11^.

We have previously demonstrated that the highly acetic acid tolerant yeast, *Zygosaccharomyces bailii*, has a unique ability to remodel the composition of its plasma membrane in response to acetic acid stress, greatly increasing the fraction of sphingolipids at the expense of glycerophospholipids^12^. Using *in silico* molecular dynamic simulations in combination with *in vivo* reduction of the sphingolipid fraction, we further demonstrated that the high sphingolipid content in the plasma membrane reduced the rate of acetic acid diffusion into the cell, resulting in increased acetic acid tolerance^8^.

Sphingolipid synthesis in *S. cerevisiae* requires three main building blocks: a long-chain base (LCB), a very-long-chain fatty acid (VLCFA, C_24_-C_26_), and an inositol-containing head group. A simplified illustration of the sphingolipid synthesis pathway is shown in Fig. 1. A LCB is formed through the condensation of serine and a fatty acyl-CoA (C_16_-C_18_) with the serine palmitoyltransferase (SPT) complex consisting of the two main proteins Lcb1 and Lcb2, and the smaller subunit Tsc3^13^,^14^. A C_18_ LCB is formed by condensation of C_16_ fatty acid with serine followed by decarboxylation. The activity of the SPT complex is negatively regulated by the two paralogues Orm1 and Orm2, through physical interaction, as well as by Sac1, which negatively regulates the complex via an unknown mechanism^15^. A VLCFA is formed by a cyclic series of elongation reactions in the endoplasmic reticulum (ER) carried out by the fatty acid elongation complex, in which Elo1, Elo2 and Elo3 are elongases with affinity for different fatty acyl chains, but only Elo3 is able to synthesize the longest C_26_ fatty acids^16^. LCBs and VLCFAs are combined into ceramides by the action of ceramide synthase, consisting of one of the paralogues Lag1 or Lac1 together with Lip1^17^. Phosphatidylinositol (PI) donates its inositol phosphate group to the ceramide moiety catalysed by the enzyme Aur1, forming inositol phosphoceramide (IPC). Mannosyl-inositol phosphoceramide (MIPC) is then formed by the addition of mannose to the inositol unit. In the last reaction step of sphingolipid synthesis, a second inositol phosphate from PI is added to MIPC, resulting in the formation of mannosyl-di-inositol phosphoceramide (MIP_2_C)^18^. IPC, MIPC and MIP_2_C are the sphingolipids located to the plasma membrane, and are collectively referred to as complex sphingolipids.

We propose membrane engineering as a tool to reduce the effects of inhibitors, and focus in the present study on the possibility of modulating the abundance of complex sphingolipids in the cell membrane of *S. cerevisiae* by overexpressing the genes *ELO3* and *AUR1* in combination with the deletion of the genes *ORM1* and *ORM2* (Fig. 1).

## Materials and Methods

### Strains and strain construction

*S. cerevisiae* strain CEN.PK 113_6B (*MATa, SUC2, MAL2-8*^*c*^, *ura3-52, leu2, trp1*)^19^ was used as the background strain. The constructed strains are listed and described in Table 1. All strains were constructed by integrating the changes into the yeast genome. The *ELO3* and *AUR1* gene sequences were amplified from genomic DNA of CEN.PK 113_6B using high-fidelity DNA polymerase (Thermo Fisher Scientific) and cloned into the integrative plasmids YIp211 and YIp128, respectively. The genes were constitutively expressed by the strong *TEF2* promoter and terminated using *CYC1*. YIp211 harboured the *URA3* marker, while YIp128 harboured the *LEU2* marker. The plasmids were linearized using the restriction enzyme Stu1 or Cla1 cleaving the *URA3* or *LEU2* gene, to allow for homologous recombination at the *ura3* or *leu2* locus of the genome. For the control strains, empty plasmids harbouring only the marker gene were integrated into the marker locus. The plasmid constructs were verified by DNA sequencing before integration.

Deletion cassettes were constructed using high-fidelity DNA polymerase to amplify the *TRP1* or KanMX marker from YIp204 or pUG6 plasmids. Primers with 20 bases binding to the marker sequence and 40 bases non-bonding tails, corresponding to the 5′ or 3′ ends respectively, of either the *ORM1* or *ORM2* gene were used. The cassettes were integrated at the locus of the gene to be deleted, looping out the gene and replacing it with the selected marker. For the control strain, only the *TRP1* gene was amplified and integrated at the *trp1* locus.

Yeast transformation was performed using the LiOAc method^20^, and transformants were selected on yeast nitrogen base (YNB) plates with complete supplement mixture (CSM) minus the appropriate dropout (6.9 g L^−1^ YNB with ammonium sulphate, 0.74 g L^−1 ^CSM-ura/leu/trp (MP Biomedicals), 20 g L^−1^ glucose, plates 20 g L^−1^ agar). Strains with the KanMX marker were selected on yeast extract peptone dextrose (YPD) plates supplemented with 0.2 g L^−1^ Geneticin (20 g L^−1^ peptone, 10 g L^−1^ yeast extract, 20 g L^−1^ glucose, 0.2 g L^−1^ Geneticin, 20 g L^−1^ agar). Correct integration into the yeast genome was verified by PCR on extracted genomic DNA. Four to six clones from each transformation were screened for growth in liquid medium, and the clone that displayed growth similar to that of the majority of the clones was selected for subsequent experiments.

### Aerobic batch cultivation

Inoculum was prepared in Erlenmeyer flasks containing minimal medium: 20 g L^−1^ glucose, 5 g L^−1^ (NH_4_)_2_SO_4_, 0.5 g L^−1^ MgSO_4_ × 7H_2_O, 3 g L^−1^ KH_2_PO_4_, 1 mL L^−1^ vitamin solution (including 25 mg L^−1^ myo-inositol, final concentration) and 1 mL L^−1^ trace element solution. Vitamin solution and trace element solution were prepared as described previously^21^. The pH was maintained by the buffering capacity of 100 mM potassium hydrogen phthalate adjusted to pH 5 using KOH. The cultures were grown under continuous shaking at 180 rpm at 30 °C and occupied a maximum of 10% of the flask volume. Exponentially growing cells from overnight culture were harvested by centrifugation at 3000 × g for 3 min at room temperature, resuspended in fresh medium and added to the cultures at an initial optical density at 600 nm (OD_600_) of 0.2. Initial strain characterization of *ELO3-OE, AUR1-OE* and *ELO3-AUR1-OE* was performed in triplicate in 500 mL baffled Erlenmeyer flasks using a medium with the same composition and at the same culturing conditions as the inoculum.

Further characterization of the *ELO3-AUR1-OE, orm1*/*2Δ* and *ELO3-AUR1-OE-orm1*/*2Δ* mutants was carried out in triplicate in bioreactors (DASGIP AG, Jülich, Germany) with a 700 mL working volume, in minimal medium, as described above, supplemented with amino acids, 0.74 g L^−1^ CSM (MP Biomedicals) and 1.05 g L^−1^ L-serine. Serine was added due to a lack of serine in the CSM amino acid mixture. The serine concentration added corresponds to the approximate serine concentration in YPD medium^22^. Antifoam (0.1 μL mL^−1^ Antifoam 204, Sigma Aldrich) was also added to the growth medium to prevent foaming. The temperature was kept at 30 °C, and the pH was maintained at 5 by the addition of KOH. A constant airflow of 1 VVM was used to sparge the bioreactors, and the dissolved oxygen tension was maintained at a minimum of 40% by adjusting the stirrer speed throughout fermentation, using 400 rpm as the initial stirrer speed. The concentrations of oxygen and carbon dioxide in the inlet gas and in the off-gas were measured with on-line gas analysers (DASGIP AG, Jülich, Germany).

### Quantification of glucose and extracellular metabolites

Samples withdrawn from bioreactor cultures were immediately filtered using 0.2 μm nylon filters. Samples were stored at −20 °C prior to analysis. Glucose and extracellular metabolites (acetate, ethanol, glycerol, pyruvate and succinate) were analysed using HPLC (Ultimate 3000, Dionex, Sunnyvale, CA, USA) together with an Aminex HPX87-H column (Bio-Rad Laboratories, Munich, Germany). 5 mM H_2_SO_4_ was used as the mobile phase, and the column temperature was maintained at 45 °C. Ethanol, glucose and glycerol were analysed using a refractive index detector (Shodex RI-101, Showa Denko, New York, NY, USA), while acetate, pyruvate and succinate were analysed using the UV detector on the Ultimate 3000 HPLC equipment, set at a wavelength of 210 nm. Six-point standard curves were used for quantification. Evaporation of ethanol was compensated for by assuming an evaporation rate of 1% ethanol per hour, of the ethanol present at each specific time point.

### Quantification of dry cell weight

Dry cell weight was determined in duplicate by filtering approximately 10 g cell broth through dry pre-weighed 0.45 μm polyethersulfone (PES) membranes and then washing with deionized water (Sartorius Stedim, Aubagne, France). The exact weight of each sample was noted, and a density of 1 g mL^−1^ was assumed for the cell broth. The membranes were dried in a microwave oven at 120 W for 15 minutes and then placed in a silica gel desiccator for a minimum of 24 hours before weighing, to ensure that no moisture remained.

### Cell viability

Cell viability was measured during mid-exponential growth by staining cells with methylene blue and counting the fraction of living cells using a microscope.

### Gene expression analysis

Cell samples (5 mL) were collected from bioreactor cultivations during mid-exponential growth, immediately placed on ice and then harvested by centrifugation at 3000 × g for 3 min at 4 °C. The cell pellets were immediately frozen in liquid nitrogen and stored at −80 °C. The RNA was extracted at a later time using the RNeasy Mini kit (Qiagen) according to the manufacturer’s instructions. Adequate integrity of the extracted RNA was confirmed by gel electrophoresis. Reverse transcription of RNA to cDNA was performed using the RevertAid H Minus First Strand cDNA Synthesis kit (Thermo Fisher Scientific) with oligo (dT)18 primers, according to the manufacturer’s instructions. Control reactions to which no reverse transcriptase had been added (no RT) were also included.

Expression levels of *ELO3* and *AUR1* were analysed using quantitative real-time PCR (qPCR) using Brilliant II SYBR Green qPCR Master Mix (Agilent Technologies) and a Stratagene MX3005P qPCR instrument (Agilent Technologies). Primers amplifying the *ELO3* and *AUR1* gene segments were designed to avoid the formation of secondary structures of the amplicons and the primers using the Primer3 software^23^,^24^, see Table 2. qPCR reactions were prepared with 2 ng μL^−1^ cDNA template and 0.3 μM of each primer in 25 μL reaction volume. DNA was amplified using the following temperature profile: initial denaturation for 10 min at 95 °C, 40 cycles of amplification with 30 s denaturation at 95 °C and 1 min annealing/elongation at 60 °C. The specificity of the amplicons was then determined by dissociation of the DNA duplex by for 1 min at 95 °C, 1 min at 55 °C and 30 s at 95 °C. Three technical replicates of each of the three biological replicates were evaluated.

The MxPro software (Agilent Technologies) was used to analyse the data. Thresholds for quantification were set in the exponential region of the amplification curve, and the program determined the quantification cycle (C_q_) based on the threshold for quantification. Expression levels of *ELO3* and *AUR1* were normalized to the expression of the *TAF10* reference gene. Expression levels of the target genes were calculated as the relative expression between the mutant and the wild type using the 2^−ΔΔCq^ method^25^. Control reactions without a template gave almost no amplification, while controls without reverse transcription (no RT) had a C_q_ value more than seven cycles higher than the C_q_ value of the target reaction, thereby indicating sufficiently low background levels to ensure no interference with the results. Four-point standard curves were used to determine the reaction efficiencies (101% for the *ELO3* gene and 98% for the *AUR1* gene), both of which are sufficient not to influence the calculated expression levels.

### Lipid analysis

Cells corresponding to an OD_600_ of 25 were collected from bioreactor cultivations during mid-exponential growth and quenched by adding 100% (w/v) trichloroacetic acid (TCA) directly to the samples to give a final TCA concentration of 5% (w/v). Quenched samples were placed on ice for 10 minutes and then centrifuged at 3000 × g for 3 min at 4 °C to obtain a cell pellet. The cell pellet was then washed with 10 mL of 5% (w/v) TCA and then with 10 mL distilled water before freezing of the pellet at −80 °C until analysis.

Lipids were extracted by re-suspending the cell pellet in 3 mL extraction solvent containing ethanol, water, diethyl ether, pyridine and 4.2 N ammonium hydroxide (15:15:5:1:0.18, v/v). Internal standards were added (3.75 nmol PC31:1, 3.75 nmol PE31:1, 3.0 nmol PI31:1, 2.0 nmol PS31:1, 0.6 nmol C17Cer and 1 nmol C8GC) and glycerophospholipids and sphingolipids were thereafter extracted as described previously^26^.

Glycerophospholipids and sphingolipids were analysed using electrospray ionization multiple-reaction-monitoring mass spectrometry (ESI-MRM-MS) using a triple-stage quadrupole mass spectrometer (TSQ Vantage MS, Thermo Scientific, Whaltman, MA, USA) equipped with a robotic nanoflow ion source (Nanomate, Advion Biosciences, Ithaca, NY, USA). The different lipid species were quantified in relation to the appropriate internal standard and normalized to the total amount of phosphate in each sample. Three to six technical replicates were analysed for each of the three biological replicates. The results are expressed as apparent quantities in arbitrary units (a.u.). The arbitrary units of glycerophospholipids are comparable within the different classes, but are not comparable with the arbitrary units of sphingolipids. The absolute quantities of the three classes of sphingolipids could not be determined as no standards were available.

### Statistical analysis

Statistical differences were tested between the constructed strains for each measured parameter. To account for multiple comparisons of strains, one-way ANOVA was selected as statistical test. To allow for pairwise comparisons, Turkey HSD was used in conjunction to the ANOVA analysis.

## Results and Discussion

**Figure 1:**
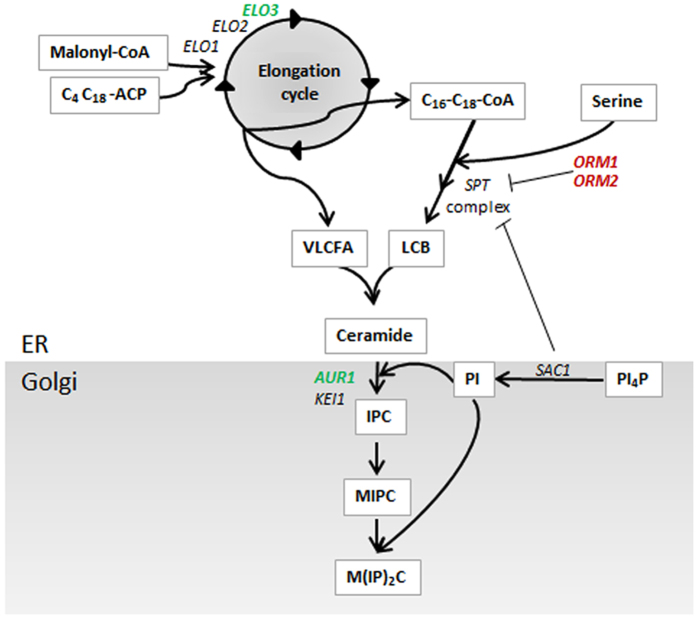
Simplified illustration of sphingolipid metabolism in *S. cerevisiae*. Genes overexpressed in this study are indicated in green and genes deleted are indicated in red. Abbreviations: VLCFA, very-long-chain fatty acid (C_24_-C_26_); LCB, long-chain base; PI, phosphatidylinositol; PI4P, phosphatidylinositol 4-phosphate; IPC, inositol phosphoceramide; MIPC, mannosyl-inositol phosphoceramide; MIP2C, mannosyl-diinositolphosphoceramide. The Illustration is drawn by the authors.

The possibility of modulating the abundance of complex sphingolipids in the cell membrane of *S. cerevisiae* towards the final goal of developing robust cell factories was investigated. To increase the flux through the sphingolipid biosynthesis pathway, VFLCA, LCB and IPC production were targeted by overexpression of the genes *ELO3* and *AUR1* in combination with the deletion of the genes *ORM1* and *ORM2* (Fig. 1). The engineered strains were evaluated in terms of cell physiology and cell lipid profile.

### Overexpression of *ELO3* and *AUR1* causes no change in the lipid profile

**Figure 2:**
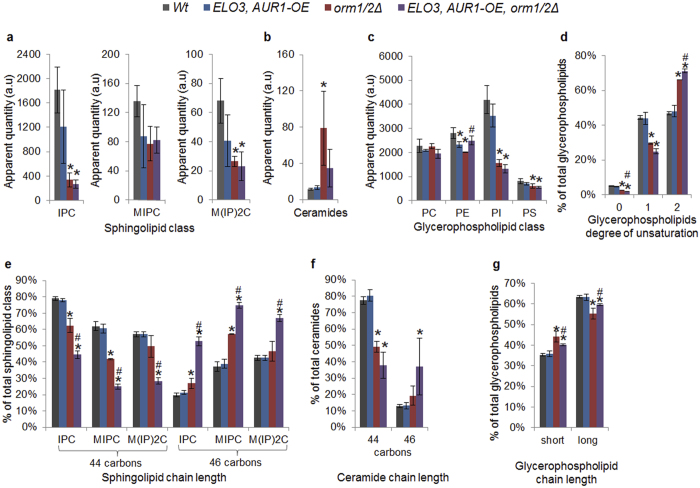
Lipid profiles of the constructed strains analysed during mid-exponential growth in minimal medium supplemented with amino acids. (**a**) Complex sphingolipid classes. (**b**) Total ceramides. (**c**) Glycerophospholipid classes. (**d**) Degree of unsaturation per lipid in total glycerophospholipids. (**e**) Complex sphingolipid total chain length per lipid. (**f**) Ceramide chain length per lipid. (**g**) Total glycerophospholipid chain length per lipid. *Significant difference compared to wild type. ^#^Significant difference compared with *orm1*/*2Δ* mutant. Differences obtained using one-way ANOVA with post-hoc Tukey HSD for multiple pairwise comparisons (*p* < 0.05). The results were calculated from three biological replicates and are given as the mean ± standard deviation.

In an attempt to increase the production of VLCFAs, the *ELO3* gene encoding a protein in the fatty acid elongation complex required for the production of C_26_ fatty acids^27^, was overexpressed. *AUR1* was also overexpressed in an attempt to increase the flux through the sphingolipid synthesis pathway, resulting in the accumulation of complex sphingolipids rather than the accumulation of the toxic intermediate ceramide. Initial characterization of the single and double mutants in aerobic shake flasks with buffered minimal medium revealed no effect on maximum specific growth rate, cell viability or metabolite profile (ethanol, glycerol, acetate, succinate, pyruvate) compared to the wild type (data not shown). Therefore, only the double mutant was selected for further characterization using pH- and oxygen-controlled bioreactors with minimal medium supplemented with amino acids. The *ELO3-AUR1-OE* mutant behaved similarly in this set-up, showing no significant effect on the fermentation profile compared to the wild type (Table 3). Investigation of the lipid profile, specifically the complex sphingolipid, ceramide and glycerophospholipid profile, revealed no significant difference between the *ELO3-AUR1-OE* mutant and the wild type (Fig. 2). To verify the overexpression of *ELO3* and *AUR1*, the RNA levels of the specific genes were quantified using qPCR, which revealed a 1.9× average ((1.6–2.4)×, 95% CI) increase in RNA levels of *ELO3*, and a 5.2× average ((4.2–6.3)×, 95% CI) increase in RNA levels of *AUR1*.

The small impact of the *ELO3-AUR1-OE* modification was partly expected, since no measures had been taken in this strain to increase the LCB production. Therefore, even with an increased capacity to produce VLCFAs, ceramide synthesis and subsequent complex sphingolipid synthesis, promoted by the overexpression of *AUR1*, could not increase due to limited LCB availability.

The effect of *ELO3* and *AUR1* overexpression on the lipid profile has not been investigated previously, although lipid data showing the reduced expression of these genes are available in the literature. Strains with *elo3Δ* produce shorter acyl chains^27^,^28^,^29^, higher ceramide levels and lower levels of total complex sphingolipids than the wild type^15^,^30^. Similarly, abolished Aur1 activity resulted in increased ceramide accumulation and reduced total levels of complex sphingolipids^31^. However, reducing the flux through a metabolic pathway can be attained by completely suppressing the enzymatic activity of certain steps in the pathway and relatively easy create a different lipid profile. On the other hand, significantly increasing the flux can be challenging, as it is crucial to identify and target the steps in the pathway that exert the highest flux control, otherwise no effect will be seen. Limited effect of the overexpression of structural genes may also be due to decoupling of mRNA levels from the amount of active enzymes, post-translational modifications altering the enzyme activity, the low availability of other interacting enzymes, or feedback inhibition of the enzyme by the downstream products. Elo3 is part of a large enzyme complex^32^ and may be regulated by phosphorylation, as shown for its homologue Elo2^29^. In addition, the activity of Aur1 has been shown to be dependent on the enzyme Kei1^33^. Further investigations are required to determine whether these factors restrict the effect of overexpression of *ELO3* and *AUR1*, or whether stronger overexpression of the genes would result in changes in the lipid profile.

### *ORM1*/*2* deletion increases ceramide levels but reduces the fraction of complex sphingolipids

The SPT complex, catalysing the formation of LCB from C_16_ and C_18_ fatty acids, is a rate-limiting enzyme complex in sphingolipid production^15^ and thus likely exerts a high flux control. Overexpression of the genes encoding enzymes in the complex will probably have a limited effect on LCB production, as they are suppressed by the negative regulators *ORM1* and *ORM2*^15^. Therefore, *ORM1* and *ORM2* were deleted in an attempt to increase the flux through the sphingolipid pathway. The resulting *orm1*/*2Δ* mutant exhibited a significantly impaired viability in aerobic shake flasks with buffered minimal medium, so a richer medium supplemented with amino acids was used to reduce the metabolic burden. The strain was cultivated in pH- and oxygen-controlled bioreactors, thereby avoiding the variation in aeration and temperature that normally occurs when shake flasks are removed from the incubator for sampling. This set-up improved the cell viability of the *orm1*/*2Δ* mutants to 89% at mid-exponential growth, while the wild type remained fully viable (Table 3). Another reason for adding amino acids to the minimal medium was to ensure that serine shortage did not limit sphingolipid production, as the SPT complex has been shown to be regulated upon serine availability^22^,^34^.

Physiological characterization of aerobic batch bioreactor cultivations revealed a 19% lower maximum specific growth rate, a 30% lower ethanol yield and similar biomass yield on glucose, when comparing the *orm1*/*2Δ* mutant to the wild type. A decrease in ethanol yield, as observed here, is usually associated with an increase in biomass yield due to elevated respiration. However, this was not observed in the *orm1*/*2Δ* mutant due to biomass loss by the lysis of non-viable cells, as indicated by the lower final biomass concentration after all the carbon sources had been depleted, and the difficulties to close the carbon balance for this specific mutant. The glycerol yield was 46% higher than in the wild type, which is a typical sign of increased cellular stress^35^.

Lipid analysis showed a decrease in all three complex sphingolipid classes (IPC, MIPC and MIP_2_C), although these were only statistically significant for IPC and MIP_2_C, which decreased by 82% and 61%, respectively, in the *orm1*/*2Δ* mutant compared to the wild type (Fig. 2a). The opposite trend was observed for the precursor ceramide, which increased on average by 680% in the *orm1*/*2Δ* mutant compared to the wild type (Fig. 2b). In addition, the glycerophospholipid class PI, required for the synthesis of complex sphingolipids decreased by 63% in the *orm1*/*2Δ* mutant compared with the wildtype, while only minor differences were observed for the other glycerophospholipid classes (Fig. 2c). Owing to the lower degree of unsaturation of PI compared to other glycerophospholipid classes, the decrease in PI also contributed to an increase in the total glycerophospholipid unsaturation level (Fig. 2d). Fatty acyl chain length increased slightly in the complex sphingolipids and ceramides, and decreased slightly in the total glycerophospholipids (Fig. 2e,f,g).

The results of previous studies on the effect of the *orm1*/*2Δ* on the lipid profile are inconsistent. Some studies have reported an increase in the ceramide content^15^,^36^, as in the present study, while others reported a decrease^37^. Furthermore, the total level of complex sphingolipids has been reported to increase^15^ as well as decrease^36^. To the best of our knowledge, no previous study has described the effect of the *orm1*/*2Δ* on the glycerophospholipid profile. Serine, used for LCB synthesis, and inositol, used for PI synthesis, are critical medium components affecting sphingolipid synthesis^22^,^37^, and a difference in medium composition could perhaps explain the discrepancy in the results reported so far. Breslow *et al*.^15^ used a complex YPD medium, Han *et al*.^37^ used a minimal medium with 36 g/L myo-inositol, Shimobayashi *et al*.^36^ used a minimal medium with 2 mg/L myo-inositol, while in the present study a minimal medium supplemented with amino acids, including 1.1 g/L serine and 25 mg/L myo-inositol, was used.

The increase in ceramide seen in the *orm1*/*2Δ* mutant, compared to the wildtype, in the present study suggests that the flux control has shifted from the SPT complex to Aur1. In addition, the decrease in complex sphingolipids indicates not only a shift of the rate controlling step but a reduced rate of the Aur1 reaction, perhaps due to low levels of PI, the second substrate of Aur1. In fact, lipid analysis showed reduced total levels of PI in the cell, although the PI concentrations in the Golgi, used by Aur1, may not mirror the total levels of PI. Instead the decrease in complex sphingolipids could be linked to a described second function of the Orm proteins in stimulating the synthesis of complex sphingolipids in the Golgi, possibly through the activation of Sac1^36^. Sac1 cycles from the ER, where it interacts physically with the Orm proteins, to the Golgi, where it catalyses the release of PI, required for the synthesis of complex sphingolipids by Aur1^38^. Perhaps the absence of Orm proteins leads to a dysfunctional Sac1, not capable of catalysing the release of PI in the Golgi. This hypothesis is supported by the fact that deletion of the *SAC1* gene significantly decreases the levels of both PI and complex sphingolipids^38^. Alternatively, the low PI and complex sphingolipid levels suggest inositol deficiency, as inositol is required for PI synthesis and the *orm1*/*2Δ* mutant has previously been shown to be an inositol auxotroph^37^. However, our medium contained 25 mg/L myo-inositol, which should be sufficient as the nutrient levels in this medium have been set so as to not limit growth to when using 2% glucose as carbon source^21^. Furthermore, commercial mineral medium (YNB, MP Biomedicals) contains only 2 mg/L myo-inositol.

### *ELO3* and *AUR1* overexpression in combination with *ORM1*/*2* deletion partly reverses the growth defect of the *ORM1*/*2* deletion mutant, but it does not increase the fraction of complex sphingolipids

Only a combined design, targeting the enzymes of the major bottlenecks in the sphingolipid biosynthesis pathway, will fully reveal the effect of the engineering strategy. The *ELO3-AUR1-OE* mutant exhibited no significant physiological or lipid profile differences compared to the wild type, although additional effects were observed in combination with *orm1*/*2Δ*. The low viability and specific growth rate of the *orm1*/*2Δ* mutant was substantially improved in the *ELO3-AUR1-OE-orm1*/*2Δ* mutant, reaching levels similar to those in the wild type (Table 3). This beneficial effect of *ELO3-AUR1-OE* on the *orm1*/*2Δ* mutant is in line with an observation from a large genetic interaction screen showing that *AUR1* was the gene among 1400 damped or deleted genes that exacerbated the effect of *orm2Δ* most^15^, as previously discussed^36^. In addition, the average ceramide level was lower in the *ELO3-AUR1-OE-orm1*/*2Δ* strain than in the *orm1*/*2Δ* mutant (Fig. 2b), suggesting an increase in downstream flux capacity due to *AUR1* overexpression, potentially explaining the increased viability of this strain, as ceramides are known inducers of apoptosis^39^. The difference in ceramide levels was, however, not statistically significant, and additional studies must be performed before any conclusions can be drawn. Interestingly, no other major differences were observed in the lipid classes between the two strains (Fig. 2c), indicating that Aur1 despite being over expressed, still exerts the highest flux control of the pathway. The shortage in PI supply capacity due to the *orm1*/*2Δ* may prevent an increase in flux in the sphingolipid pathway in the *ELO3-AUR1-OE-orm1*/*2Δ* mutant.

When investigating the sphingolipid and glycerophospholipid chain length in detail, the *ELO3*-*AUR1-OE-orm1*/*2Δ* mutant showed an increase in both complex sphingolipid and glycerophospholipid chain length compared to the *orm1*/*2Δ* mutant – a difference which was not observed when comparing *ELO3*-*AUR1-OE* with the wild type (Fig. 2e,g). The increase in the sphingolipid classes was observed specifically in the percentage of C_46_ species present. The ceramide chain length profile showed the same trend, but this was not statistically significant (Fig. 2f). Sphingolipid chains with 44 carbons are most likely a combination of a C_18_ LCB and a C_26_ fatty acid, as this combination has previously been shown to be the most common sphingolipid species^30^. Synthesis of fatty acids longer than C_26_ have not been reported. Sphingolipid chains with 46 carbons should hence correspond to C_20_/C_26_ species, suggesting a fatty acid shift in the *ELO3*-*AUR1-OE-orm1*/*2Δ* mutant from C_16_ to C_18_, which is the fatty acid that gives rise to C_20_ LCB. This observed shift in fatty acids may thus be the effect of increased elongation in the fatty acid elongation complex due to the overexpression of *ELO3*.

### General discussion

In our previous studies, on the membrane properties of *Z. bailii*^8^,^12^, we suggested the potential of membrane engineering as a tool to create more robust microbial cell factories. Engineering the cell to achieve specific changes in the lipid composition of the membrane may, however, prove to be a challenging task. The aim of the present study was to increase the fraction of complex sphingolipids in the cell membrane of *S. cerevisiae* by altering the expression of genes associated with the production of LCBs and VLCFAs, and the conversion of ceramide into complex sphingolipids. Approaches such as this, where several components of the sphingolipid metabolism are targeted simultaneously, have so far been rare. Most previous knowledge regarding sphingolipid metabolism has been obtained through investigations of a single step in the pathway or in large genetic screens^40^ with the aim of understanding genetic interactions, rather than changing the lipid profile. In the present study, we were able to demonstrate that combining *ELO3-AUR1-OE* with *orm1*/*2Δ* reduces the stress caused by the *orm1*/*2Δ* leading to improved cell growth and fermentative performance, as well as changes in fatty acyl chain length. However, the strategy did not result in an increase in complex sphingolipids, and can therefore not be used as a means of increasing microbial tolerance to inhibitory compounds. Importantly, we were able to gain new insights into the sphingolipid metabolism of *S. cerevisiae* that could aid in the design of new and more efficient engineering strategies.

The flux of a metabolic pathway is dependent on the amount and activity of the enzymes catalysing each step and the available metabolite concentrations. Difficulties may be encountered in strategies to increase the amount of enzymes available to catalyse a specific step on a genetic level, as in the present study, due to transcriptional regulation altering mRNA levels and post-translational regulation altering enzyme activity. Sphingolipid biosynthesis has recently been shown to be strongly regulated by phosphorylation^41^, so a strategy targeting regulatory enzymes instead of structural enzymes may be more favourable, although such a strategy would be difficult to design due to our, as yet, incomplete understanding of regulation of the sphingolipid metabolism^42^. The double role played by sphingolipids in the cell, as structural building blocks in the cell membrane, and as signalling molecules involved in many cellular functions, further complicates the picture^42^. The main role of some enzymes is to modify sphingolipid concentrations for signalling purposes, and these may not be involved in regulating the bulk concentration of complex sphingolipids, as they are located in different compartments^43^. Furthermore, a comprehensive design targeting all the critical steps in the pathway is required to ensure that substrate is available for each of the individual biosynthetic steps. In sphingolipid metabolism, it is also essential to maintain the correct balance between pathway intermediates such as VLCFAs, LCBs and ceramides to avoid the toxic effects of these intermediates at elevated levels^44^. In mammalian cells, the ceramide transport protein (CERT) is essential to relieve the stress induced by high ceramide levels^45^. CERT transports ceramides produced in the ER to the Golgi, where they are used for the production of complex sphingolipids. A similar function was recently found in yeast by Prinz and co-workers, who discovered that Nvj2 had a ceramide transfer function which, when overexpressed, relieved the ceramide-induced stress in *S. cerevisiae*^46^. For future engineering strategies we suggest the combination of *NVJ2* overexpression and *AUR1* overexpression to ensure that ceramides in the ER are available in the Golgi for use by Aur1 to synthesize complex sphingolipids. Finally, membrane sphingolipids can be in fact considered as a metabolic intermediate rather than a final product, as they are recycled by the cell^47^. Therefore, sphingolipid recycling reactions can be also considered as a potential target.

Difficulties in increasing pathway flux due to post-translational regulation and keeping toxic pathway intermediates sufficiently low, led us to suggest an alternative strategy, namely one aimed at introducing a pathway bottleneck elsewhere in lipid metabolism. This strategy relies on the fact that the cell itself redirects the flux to increase the desired group of lipids, in order to synthesize sufficient levels of lipids to maintain a functional plasma membrane. This strategy may seem farfetched, but reducing the flux not only limits the problems associated with unexpected post-translation modification, but it is also significantly more straightforward to construct a single rate-controlling step than to achieve increased flux through the entire metabolic pathway. For example, enzymes catalysing glycerophospholipid production could be downregulated to increase the fraction of complex sphingolipids in the cell, mimicking the response to an inhibitory concentration of acetic acid observed in *Z. bailii*, where a considerable decrease in glycerophospholipid was compensated by a high increase in complex sphingolipids^12^.

To prove the value of membrane engineering, an interesting alternative to modification of the native lipid production pathways of the cells could be membrane alteration using heterologous lipids, thereby circumventing cellular regulation. Archaea lipids and their properties have been studied extensively, as reviewed by Matsumi *et al*.^48^, and the introduction of trans fatty acids into *E. coli* has recently been shown to successfully increase its tolerance to many alcohols, aromatic solvents and organic acids^11^.

## Conclusions

In an attempt to increase the amount of complex sphingolipids in *S. cerevisiae, ELO3* overexpression, *AUR1* overexpression and *ORM1*/*2* deletion were performed. Neither *ELO3* nor *AUR1* overexpression influenced the lipid metabolism in our set-up, while *orm1*/*2Δ* instead caused a decrease in both complex sphingolipids and PI glycerophospholipids. However, by combining *ELO3-AUR1-OE* with the *orm1*/*2Δ*, the *ELO3-AUR1-OE* background alleviated the reduction in growth caused by the *orm1*/*2Δ*, and increased the fatty acyl chain length, demonstrating that overall approaches combining alterations in several steps in the pathway may give additional effects not observed with the single mutations. We suggest that our findings can be used as the foundation for further studies on sphingolipid metabolism. Furthermore, to begin evaluating the potential of membrane engineering, we recommend that changes are made in the cell membrane that can be achieved by altering only a few genes, or the introduction of new lipid species by the expression of genes encoding heterologous synthesis pathways in order to avoid cellular regulation.

## Additional Information

**How to cite this article:** Lindahl, L. *et al*. Membrane engineering of *S. cerevisiae* targeting sphingolipid metabolism. *Sci. Rep.* **7**, 41868; doi: 10.1038/srep41868 (2017).

**Publisher's note:** Springer Nature remains neutral with regard to jurisdictional claims in published maps and institutional affiliations.

## Figures and Tables

**Table 1:** Strains constructed in this study.

Background strain	Recombinant strain	Genotype
CEN.PK 113-6B	*wt*	*MATa, SUC2, MAL2-8*^*c*^, *ura3-52::URA3, leu2::LEU2, trp1::TRP1*
CEN.PK 113-6B	*ELO3-OE*	*MATa, SUC2, MAL2-8*^*c*^, *ura3-52::URA3-TEF2p-ELO3-CYC1t, leu2::LEU2, trp1::TRP1*
CEN.PK 113-6B	*AUR1-OE*	*MATa, SUC2, MAL2-8*^*c*^, *ura3-52::URA3, leu2::LEU2-TEF2p-AUR1-CYC1t, trp1::TRP1*
CEN.PK 113-6B	*ELO3-AUR1-OE*	*MATa, SUC2, MAL2-8*^*c*^, *ura3-52::URA3-TEF2p-ELO3-CYC1t, leu2::LEU2-TEF2p-AUR1-CYC1t, trp1::TRP1*
CEN.PK 113-6B	*orm1*/*2Δ*	*MATa, SUC2, MAL2-8*^*c*^, *ura3-52::URA3, leu2::LEU2, trp1, ORM1::TRP1, ORM2::kanMX*
CEN.PK 113-6B	*ELO3-AUR1-OE-orm1*/*2Δ*	*MATa, SUC2, MAL2-8*^*c*^, *ura3-52::URA3-TEF2p-ELO3-CYC1t, leu2::LEU2-TEF2p-AUR1-CYC1t, trp1, ORM1::TRP1, ORM2::kanMX*

**Table 2:** Primers for gene expression analysis.

Gene	Primer	Sequence 5′-3′
*AUR1*	AUR1 qRT-PCR fwd	ACTAATCCCGCACCTTGGAT
	AUR1 qRT-PCR rev	CATGTTAGGATGGGCAAGGC
*ELO3*	ELO3 qRT-PCR fwd	TGGCTAACGGGTATCATGCT
	ELO3 qRT-PCR rev	AATGGAGAGGCGTTCAAGGC
*TAF10*	TAF10 qRT-PCR fwd	TACCCGAATTTACAAGAAAAGATAAGA
	TAF10 qRT-PCR rev	ATTTCTGAGTAGCAAGTGCTAAAAGTC

**Table 3:** Physiological parameters obtained from aerobic batch fermentation in mineral medium supplemented with amino acids.

Strain	Physiological parameters					
	μ_max_ (h^−1^)	Viability (%)	Final biomass^1^ (g dw/L)	Y_Biomass/substrate_ (cmol × cmol^−1^)	Y_EtOH/substrate_ (cmol × cmol^−1^)	Y_Glycerol/substrate_ (cmol × cmol^−1^)
*wt*	0.44 ± 0.00	100 ± 0	5.0 ± 0.1	0.12 ± 0.00	0.43 ± 0.01	0.013 ± 0.000
*ELO3, AUR1-OE*	0.44 ± 0.01	100 ± 0	5.1 ± 0.1	0.12 ± 0.00	0.44 ± 0.04	0.012 ± 0.000
*orm1*/*2Δ*	0.37 ± 0.03*	89 ± 1*	3.9 ± 0.1*	0.13 ± 0.01	0.33 ± 0.04*	0.019 ± 0.000*
*ELO3, AUR1-OE, orm1*/*2Δ*	0.42 ± 0.00^#^	97 ± 2	4.6 ± 0.0*^#^	0.14 ± 0.01*	0.40 ± 0.01^#^	0.014 ± 0.000^#^

^1^Final biomass concentration when all carbon sources had been depleted, measured in gram dry weight per litre culture. Results were calculated from three biological replicates and are given as the mean ± standard deviation. Differences obtained using one-way ANOVA with post-hoc Tukey HSD for multiple pairwise comparisons (p < 0.05). *Significant difference compared to wild type, ^#^Significant difference compared to orm1/2Δ.
